# Peanut Leaf Wilting Estimation From RGB Color Indices and Logistic Models

**DOI:** 10.3389/fpls.2021.658621

**Published:** 2021-06-18

**Authors:** Sayantan Sarkar, A. Ford Ramsey, Alexandre-Brice Cazenave, Maria Balota

**Affiliations:** ^1^School of Plant and Environmental Sciences, Virginia Tech, Tidewater AREC, Suffolk, VA, United States; ^2^Department of Agricultural and Applied Economics, Virginia Tech, Blacksburg, VA, United States

**Keywords:** peanut leaf wilting, RGB color space indices, logistic regression, machine learning, high-throughput phenotyping

## Abstract

Peanut (*Arachis hypogaea* L.) is an important crop for United States agriculture and worldwide. Low soil moisture is a major constraint for production in all peanut growing regions with negative effects on yield quantity and quality. Leaf wilting is a visual symptom of low moisture stress used in breeding to improve stress tolerance, but visual rating is slow when thousands of breeding lines are evaluated and can be subject to personnel scoring bias. Photogrammetry might be used instead. The objective of this article is to determine if color space indices derived from red-green-blue (RGB) images can accurately estimate leaf wilting for breeding selection and irrigation triggering in peanut production. RGB images were collected with a digital camera proximally and aerially by a unmanned aerial vehicle during 2018 and 2019. Visual rating was performed on the same days as image collection. Vegetation indices were intensity, hue, saturation, lightness, a^∗^, b^∗^, u^∗^, v^∗^, green area (GA), greener area (GGA), and crop senescence index (CSI). In particular, hue, a^∗^, u^∗^, GA, GGA, and CSI were significantly (*p* ≤ 0.0001) associated with leaf wilting. These indices were further used to train an ordinal logistic regression model for wilting estimation. This model had 90% accuracy when images were taken aerially and 99% when images were taken proximally. This article reports on a simple yet key aspect of peanut screening for tolerance to low soil moisture stress and uses novel, fast, cost-effective, and accurate RGB-derived models to estimate leaf wilting.

## Introduction

**FIGURE 1:**
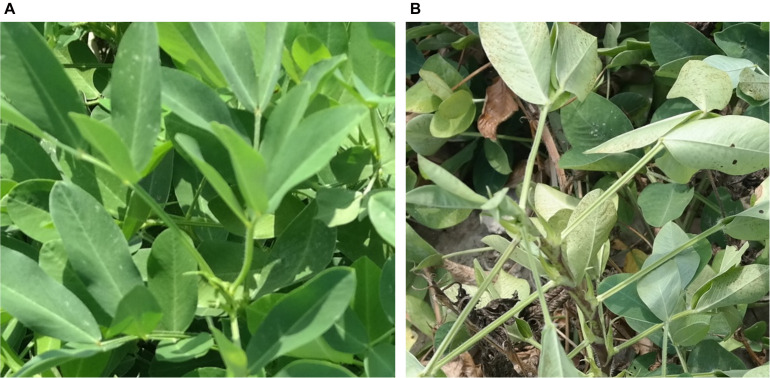
**(A)** Close up picture of a healthy plant, which has greener, front-facing leaves and no bare ground visible as compared with **(B)** a wilted plant with lighter green leaves and bare ground visible. This visual difference can be exploited and used to quantify wilting using pictures.

Peanut is an important oil and food crop grown on 28 million hectares (ha) worldwide (FAO STAT, 2020). In the United States, peanut provides relatively high net returns for growers; it is grown annually on approximately 619,000 ha in 11 states with an average production of 4400 kg ha^–1^ (US Department of Agriculture-National Agricultural Statistics Service, 2019). Biotic and abiotic stresses are major constraints to peanut production in all peanut growing regions. For example, in a subhumid region, such as Virginia and the Carolinas, the summer (May through October) precipitation is regularly between 500 and 1000 mm. Peanut grows on sandy soils, and in southeast Virginia, where peanut is grown, available water capacity in the first 25 cm of the soil profile varies from 0.10 to 0.15 cm/cm. From May to June, precipitation is intense, and consequently, biomass grows fast. July and August are the hottest months, and peanut is at the beginning flowering stage in early July when a combination of abundant biomass, intense water evaporation from sandy soils, and absence of precipitation can induce drought and wilting in 10 days. Under these conditions, moderate drought can reduce economic return by 20% and severe drought by 60% (Balota et al., 2020). In this environment, drought usually occurs in midseason, i.e., abundant flowering, pegging, and pod growth (July and August), not at terminal growth (September and October). It also happens fast, and plants do not translocate from older to younger leaves like cereals do. This is why peanut does not senesce under drought, likely severely wilts, ceases growth, and recovers at the first rain as in Figure 1. At the same time, high relative humidity and abundant morning dew are important water sources for peanut during water stress time as suggested in other sandy ecosystems (Liu et al., 2020). Low soil moisture stress during the flowering and pegging growth stages causes severe reduction of pod yield (Stansell et al., 1976; Pahalwan and Tripathi, 1984; Naveen et al., 1992; Smartt, 1994; Rucker et al., 1995; Prasad et al., 1999; Reddy et al., 2003; Shao et al., 2008). Drought stress during the pod and seed filling growth stages results in small and immature seeds with reduced germination, vigor, embryo membrane integrity, and embryo RNA content (Reddy et al., 2003). Calcium, usually applied at the beginning of pegging in early July, is a critical nutrient for peanut seed development; it needs to be dissolved in soil solution for absorption by pods and seeds in the ground. Low soil moisture during pod development results in calcium deficiency causing undeveloped seeds (or “pops”) and embryo damage (Skelton and Shear, 1971; Wright et al., 1991). Low soil moisture during seed maturation results in a decreased oleic to linoleic fatty acid ratio, which reduces the storage shelf life and nutritional qualities of peanuts (Hashim et al., 1993). Low moisture is responsible for reduced nodulation and nitrate reductase activity affecting N fixation and uptake and biomass and yield production (Lenka and Mishra, 1973; Kulkarni et al., 1988; Devries et al., 1989). Low moisture stressed peanuts are prone to *Aspergillus flavus* mold contamination, known to produce the carcinogenic aflatoxin (Wilson and Stansell, 1983; Sanders et al., 1993; Luis et al., 2020). When available, supplemental irrigation can ameliorate low moisture stress, but this increases input costs. For example, contacts with growers in Virginia estimate an irrigation cost of $1.24/ha and mm water (Balota, personal communication). From planting to harvest, peanut requires weekly amounts of 25–50 mm of water (Rowland et al., 2012; Putnam et al., 2014). This suggests a weekly irrigation cost of $62 per ha. In 2020, numerous peanuts fields in southeast Virginia needed irrigation every week in July to ensure adequate water supply for the crop (Balota, personal communication).

Under drought conditions, cultivars with tolerance to low moisture stress are required. Previous studies suggest that breeding selection using physiological characteristics is a better option than being selection for yield alone (Kiniry et al., 2005; Nigam et al., 2005; Nigam and Aruna, 2007; Arunyanark et al., 2008). The use of remote sensing for phenotyping is also reported (Raju et al., 2016; Reynolds and Langridge, 2016; Sreeman et al., 2018). Studies suggest that traditional breeding methods are slow, and use of remote sensing is required for faster and more accurate phenotyping (Zaman-Allah et al., 2015; Reynolds and Langridge, 2016; Sreeman et al., 2018). Aerially derived plant wilting from low-cost cameras and RGB indices could be used to select breeding lines with drought tolerance. If directly associated with yield, aerially derived wilting could be used to trigger irrigation in peanut production, similar to how remotely sensed canopy temperature has been used to trigger irrigation in Texas (Evett et al., 1996). This ensures economical water use as suggested in similar applications in Georgia (Rowland et al., 2012).

Tolerance to low soil moisture is associated with deep rooting patterns of peanut plants. Deep roots uptake water from a lower soil horizon during drought stress (Fukai and Cooper, 1995; Henry et al., 2011; Comas et al., 2013). Accurately measuring rooting depth is difficult and labor intensive with current methods (Courtois et al., 2013; Krišāns et al., 2020). Leaf wilting, however, is easily visible and represents the phenotypic expression of plants when roots cannot replenish the water lost through transpiration. Water-deficient cells have low cellular turgor pressure, causing the leaf to lose structural integrity. This causes the leaves to either fold, roll, or droop down (Blum, 2011). These symptoms constitute visual expression of wilting and can be used as a proxy for low moisture stress in plants. Studies show that reduced leaf water potential due to low moisture stress is directly related to wilting severity (Engelbrecht et al., 2007). As illustrated in Figures 1A,B, non-wilted plants have leaves facing upward with no stems or bare ground visible compared with wilted plants with folded leaves. Because, in peanut, wilting is a clear visual symptom of low moisture stress, quantifying wilting is recommended as an important step toward development of cultivars with tolerance to low soil moisture (Luis et al., 2016). Visual rating is an important tool to quantify leaf wilting in plants (Engelbrecht et al., 2007; Hamidou et al., 2012; Luis et al., 2016; Balota and Oakes, 2017; Zhou et al., 2020). Visual rating is based on morphological changes of leaves when cells become less turgid (Figure 1). However, rating thousands of plots is time-consuming and subject to human error (Milberg et al., 2008; Borra-Serrano et al., 2018). Fast and accurate methods are required to intensify phenotyping for drought tolerance in breeding programs.

Remote sensing can be used to quantify wilting based on the changes in the leaf physiology and morphology. Red, near infrared, and infrared imagery helped with development of an abundance of vegetation indices, e.g., normalized difference vegetation index (NDVI), green based NDVI (g-NDVI), red–green ratio index (RGRI), normalized green-red difference index (NGRDI), and normalized sunlit shaded index (NSSI). They were used to estimate low soil moisture stress in several agronomic and horticultural crops, including wheat (*Triticum aestivum* L.) (Oakes et al., 2020), sorghum [*Sorghum bicolor* (L.) Moench] (Sadeghpour et al., 2017), maize (*Zea mays* L.) (Wenting et al., 2014), barley (*Hordeum vulgare* L.) (Romer et al., 2012), soybean [*Glycine max* (L.) Merr.] (Zhou et al., 2020), potato (*Solanum tuberosum* L.) (Zakaluk and Ranjan, 2008), and spinach (*Spinacia oleracea* L.) (Raza et al., 2014). In these studies, multispectral and hyperspectral sensing from proximal and unmanned aerial vehicle (UAV) images were used. These expensive technologies require a high level of technical knowledge (Moshou et al., 2004).

Alternative methods derived from relatively low-cost RGB digital cameras were also developed for high-throughput phenotyping (HTP) of important agronomic crops but not for peanut (Diéguez-Uribeondo et al., 2003; Reyniers et al., 2004; Graeff et al., 2006; Mirik et al., 2006; Su et al., 2020; Zhou et al., 2020). Low-cost technology coupled with open-source computer software seem to offer advantages to more complex technologies when deriving vegetation indices capable of assessing the effect of abiotic stress on crop canopy (Casadesús et al., 2007; Casadesus and Villegas, 2014). Vegetation indices can be derived from RGB color space indices, which represent international standards for color perception by the human eye and were adopted by the Commission Internationale de l’Eclairage (CIE) in 1976 (Yam and Papadakis, 2004; Trussell et al., 2005; Casadesús et al., 2007; Liu et al., 2011; Kipp et al., 2014; Zhou et al., 2015). Several studies show that RGB color space indices, such as hue, a^∗^, u^∗^, and other derived indices, such as green area (GA) and the normalized difference CIELab index (NDlab), outperformed spectral indices such as NGRDI, NDVI, and gNDVI, in predicting yield of wheat and maize more accurately and had higher broad sense heritability for drought tolerance in forage grasses (Kefauver et al., 2015; Vergara-Díaz et al., 2015, 2016; Zhou et al., 2015; Gracia-Romero et al., 2017, 2018; Buchaillot et al., 2018, 2019; Fernandez-Gallego et al., 2019; De Swaef et al., 2021).

Our preliminary research suggests that RGB color space indices and derived vegetation indices may be suitable for estimation of leaf wilting, plant population, and pod yield of peanut (Balota and Oakes, 2016, 2017; Oakes and Balota, 2017). This study represents an in-depth analysis of the visual differences among healthy and wilted peanut plants and provides accurate machine learning models for estimation of peanut leaf wilting.

## Materials and Methods

### Experimental Design

**FIGURE 2:**
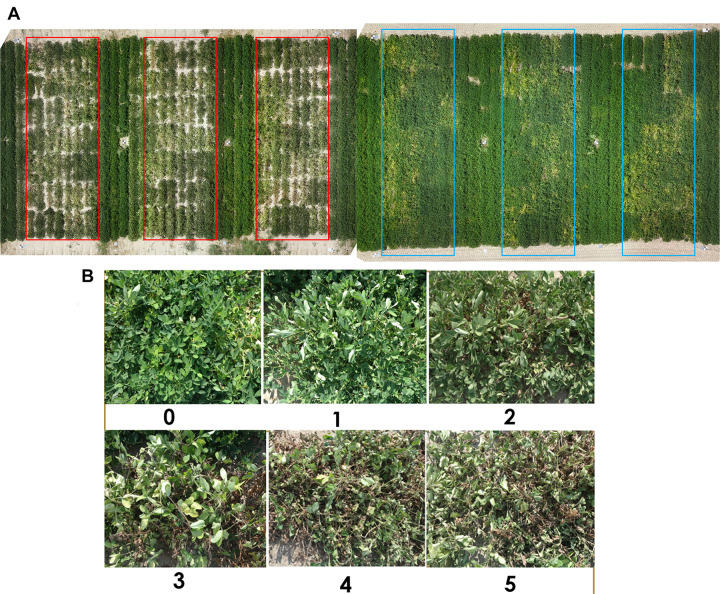
**(A)** Aerial view of the whole study area with red boxes showing three reps of the moisture-stressed plots and blue boxes showing the well-watered plots. A total of 168 (2 water regimes × 3 replications × 28 genotypes) experimental plots were used in this study. The image was taken at 14 weeks after planting. **(B)** The wilting scale used for wilting rating for peanut. The scale ranges from 0 to 5, 0 – potentially healthy plant with no wilting or leaf drooping symptoms; 1 – some terminal and newer leaf fold-up, but overall the plant looks healthy; 2 – almost all leaves fold up and show signs of wilting, lower and older leaves start to fold; 3 – wilting and drooping shows up on all leaves of the plant, low-moisture effect on older leaves is more prominent, bare ground starts to show up under the plant as leaves wilt; 4 – all leaves are wilted, and some leaves start to change color due to chlorophyll degradation, bare ground is prominently visible, some leaves have dried and crisped up; 5 – all leaves have severely wilted and color of all leaves is light green to yellow, bare ground is fully visible, more than 50% of leaves are crisp and dry, the plant is almost physiologically dead.

The experiment was performed at the Virginia Tech Tidewater Agricultural Research and Extension Center (TAREC) in Suffolk, VA, United States (latitude 36.66 N, longitude 76.73 W). Twenty-eight peanut genotypes with diverse morphological and physiological characteristics from the United States mini-core collection were selected for this study (Holbrook et al., 1993). They were planted on May 17, 2018, and April 30, 2019, in double row plots, 2.13 m long and 1.83 m wide, with a 14 seed m^–2^ seeding rate. A randomized complete block design (RCBD) with six blocks/replications was used. Each block was 21.3 m long by 7.3 m wide with five peanut border rows in between, spaced at 0.9 m (4.75 m total in between the blocks) (Figure 2A). The land was tilled and seed beds were uniformly raised to 15 cm height before planting. Cultural practices were performed as recommended by the Virginia Peanut Production Guide (Balota et al., 2020). The plots were rainfed until 8 weeks after planting (WAP). During this time, a cumulative rainfall amount of 210 mm in 2018 and 260 mm in 2019 was available to the plants. Rain-exclusion shelters were placed over three blocks (one rain-exclusion shelter over each block or replication) on July 16, 2018, and July 7, 2019, to obstruct rain and induce low soil moisture conditions. Water was kept out of the plots for 6–7 weeks before removing the rain-out shelters on August 30, 2018, and August 29, 2019.

### Ground Truth Data

Leaf wilting was scored visually using a 0–5 rating scale in both years. A score of 0 describes a potentially healthy plant with no wilting or leaf drooping symptoms; 1 describes some terminal and newer leaf fold up, but overall the plant looks healthy; 2 describes plants with almost all leaves folded up and showing signs of wilting, lower and older leaves start to fold; 3 describes wilting and drooping on all leaves of the plant, low-moisture effect on older leaves is more prominent, bare ground starts to show up under the plant as leaves wilt; 4 describes plants with all leaves wilted and some leaves starting to change color due to chlorophyll degradation, bare ground is prominently visible, some leaves have dried and crisped up; 5 describes plants with all leaves severely wilted and color of all leaves becoming light green to yellow, bare ground is fully visible, more than 50% of leaves are crisp and dry, the plants are almost physiologically dead (Figure 2B; Luis et al., 2016). Wilting was scored biweekly, from 4 until 20 WAP. In addition, the 0–5 rating scale of wilting was also converted to a binary scale of “turgid” and “wilted.” The binary rating of wilting involved classification of 0 and 1 wilting scores as turgid and scores 2–5 as wilted.

Soil moisture at 10, 20, 30, and 40 cm depth was monitored under each shelter in the plots where the “Wynne” check cultivar was grown. Every other week starting at 4 WAP, soil moisture data was extracted using a Delta-T HH2 moisture meter (Delta-T Devices Ltd., Cambridge, United Kingdom). At harvest maturity, pod yield was measured and adjusted to 7% seed moisture for individual plots. For this study, soil moisture and pod yield were collected in 2018 only because data from 2019 was for validation of models.

### Image Data

**FIGURE 3:**
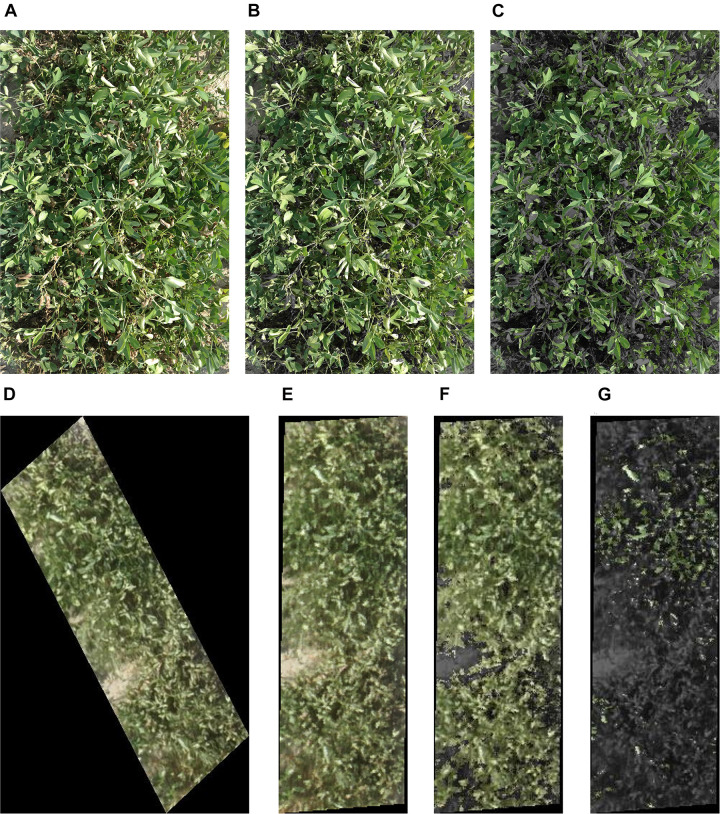
**(A)** A peanut row used for proximal RGB color and vegetation indices extraction; the picture was taken at a height of 1.2 m. **(B)** The same row after green area (GA) (pixels in 60°–120° hue angle in CIE-Lab) extraction. **(C)** The same row after greener area (GGA) (pixels in 80°–120° hue angle in CIE-Lab) extraction. **(D)** Aerial image of a single row of peanut plot taken from a height of 20 m. The row was cropped from the orthomosaic for RGB color indices extraction. **(E)** The same row as in picture ‘D’ after rotation, **(F)** extraction of GA, and **(G)** GGA. Figures **(A–C)** are 1.52 m in length and 0.91 m in width, and figures **(D–G)** are 2.13 m in length and 0.91 m in width.

Images were collected around the same day as the leaf wilting score. Proximal RGB images were collected twice within the 6 weeks of induced low moisture stress, on August 2 and 15, 2018. Aerial images were collected immediately after retracting the rain-out shelters and before a rain event on August 30, 2018. Proximal images of individual rows within each plot were taken twice, August 3 and 15, 2018, during shelter coverage from a height of 1.2 m using a Samsung NX300 digital camera (20.3 megapixel, autofocus mode, no zoom) (Figure 3A and Table 1). In 2019, images were taken proximally on August 27 and aerially on August 29 for validation of the 2018 models. Aerial images were taken with a Sony^®^ α6000 camera (24.3-megapixel, autofocus mode, no zoom) mounted on an octocopter UAV platform (model AscTec^®^ Falcon 8; Ascending Technologies, Germany). Proximal images were collected from 1100 to 1300 h on sunny days, and the aerial images were collected at noon within 6 min on sunny days as described by Sarkar et al. (2020). The flight campaign was based on waypoint navigation. The UAV was flown on autopilot at 20 m altitude with image overlap of 75% forward and 90% sideways. Flight campaign was created in AscTec^®^ Navigator 3.4.5 software (Ascending Technologies, Germany). The UAV used its built-in GPS (accuracy within 20 cm) to navigate, acquire nadir images, and coordinate recording of individual images. Images were processed into an orthomosaic using Pix4Dmapper Version 4.2.26 software (Prilly, Switzerland) to create an RGB field map. The orthomosaic was rotated to have the peanut rows perpendicular to the plane. Each individual row was cropped automatically using a precreated fishnet in the ArcMap (version 10.6) tool of the ArcGIS (ESRI, Redlands, CA, United States) (Figure 3D). The cropped rows were saved in.jpeg format, for further use in the Breedpix software (see section “Extraction of RGB Color Indices”).

**TABLE 1:** Date of proximal and aerial RGB and wilting data collection for the study.

	Image acquisition date	Type	Height	*N*	Wilting rating date
Model training	August 3, 2018	Proximal	1.2 m	84	August 1, 2018
	August 15, 2018	Proximal	1.2 m	84	August 14, 2018
	August 30, 2018	Aerial	20 m	168	August 27, 2018
Model validation	August 27, 2019	Proximal	1.2 m	168	August 30, 2019
	August 29, 2019	Aerial	20 m	168	August 30, 2019

*The ground truth date is the date of manual wilting rating for the plots. *N* = number of experimental plots used for measurement.*

### Extraction of RGB Color Indices

Proximal and aerial RGB images were used to extract 11 RGB color indices (Table 2) using the Breedpix 0.2 option from the CIMMYT maize scanner 1.16 plugin^1^ (Copyright 2015 Shawn Carlisle Kefauver, University of Barcelona; produced as part of Image J/Fiji (open source software^2^) (Schindelin et al., 2012; Rueden et al., 2017). The indices extracted were intensity, hue, saturation, lightness, a^∗^, b^∗^, u^∗^, v^∗^, GA, GGA, and CSI. The pixel selection for GA and GGA extraction is exemplified in Figures 3B,C for proximal images and Figures 3F,G for aerial images. These indices were selected because they were developed for breeding selection and successfully used in wheat and corn to predict plant biomass, leaf area index, and nitrogen content (Casadesús et al., 2007; Casadesus and Villegas, 2014; Kefauver et al., 2015).

**TABLE 2:** RGB color indices derived from proximal and aerial images using Breedpix 2.0 software.

RGB color indices	Basis of derivation
Intensity	Measures intensity or grayness in 0 (black) to 1 (white) scale in Hue Saturation Intensity (HSI) color space^1^.
Hue	Color judgment based on position in HSI color space [0°–360° (0°-red; 60°-yellow; 120°-green; 240°-blue)]^1^
Saturation	Measures dilution of pure color (hue) with white light in HSI color space (ranges from 0 to 1)^1^
Lightness	Light reflected by a non-luminous body [0 (black) to 100 (white) scale]^2^
a	Measures color shift from green (−a) to red (+a) in CIE-Lab ^†^ color space^2^
b	Measures color shift from blue (−b) to yellow (+b) in CIE-Lab color space^2^
u	Measures color shift from green (−a) to red (+a) in CIE-Luv^†^ color space^2^
v	Measures color shift from blue (−b) to yellow (+b) in CIE-Luv color space^2^
Green area (GA)	Percentage of pixels in 60°–120° hue angle in CIE-Lab^3^
Greener area (GGA)	Percentage of pixels in 80°–120° hue angle in CIE-Lab^3^
Crop senescence index	100 × (GA-GGA)/GA^4^

*^1^ Welch et al., 1991*

*^2^ Schanda, 2007*

*^3^ Casadesús et al., 2007*

*^4^ Kefauver et al., 2017*

^†^L represents Lightness in both CIE-Lab and CIE-Luv, and a* and u* represent the red-green spectrum of chromaticity and b* and v* represent yellow-blue color spectrum (Schanda, 2007).

### Rotation of Aerial RGB Images

Because the plots were north aligned (hence, not perpendicular to the plane), initially the cropped aerial images were surrounded by a black hallow (Figure 3D). To remove the hallow, the orthomosaic was rotated perpendicular to the plane in ArcGIS before rows were cropped as shown in Figure 3E. If using an unrotated orthomosaic, the black hallows around each .*jpeg* extracted image introduced error to the data. For example, the mean values of intensity, hue, saturation, lightness, a^∗^, b^∗^, u^∗^, v^∗^, GA, GGA, and CSI changed from 0.15, 67.24, 0.24, 16.93, −6.36, 19.80, −0.47, 9.75, 0.29, 0.08, and 75.98, respectively, for the oblique images to 0.37, 67.20, 0.24, 43.87, −9.30, 23.99, −1.46, 27.17, 0.68, 0.18, and 78.30 on the rotated perpendicular images.

### Statistical Analysis

#### Correlation

The association between plant wilting and the RGB color indices was assessed from the Pearson’s correlation matrix using Proc CORR in Statistical Analysis Software (SAS) 9.4 (SAS Institute Inc., Cary, NC, United States). ANOVA from Proc GLM in SAS 9.4 was used to evaluate the effect of plant wilting on pod yield, and yield means corresponding to the wilting scores 0–5 were separated using Fisher’ s least significant difference (LSD) at 5% probability.

#### Training Logistic Models

Logistic regression was used for model training because the wilting scores were discrete data. Proc LOGISTIC was used to train four models for plant wilting estimation: model 1 from proximal, model 2 from aerial indices using ordinal regression, model 3 from proximal, and model 4 from aerial indices using binary regression (Allison, 2012; Hosmer et al., 2013). Data from the 2018 study were used for model training. Models 1 and 3 included both the proximal imagery data sets. Ordinal logistic regression was used for 0–5 wilting scores because the scores were ordered from 0 to 5, whereas binary logistic regression was used for the turgid/wilted wilting score because the scores were in binary form of either turgid or wilted (Harrell, 2015a,b). Stepwise selection was used to select the best predictors, i.e., color space and vegetation indices, for the models. The Akaike information criterion (AIC) and Schwarz criterion (SC) were used to select the models with best fit in-sample, i.e., indices with the lowest AIC and SC. The AIC and SC of the model with selected predictors (selected model) were compared with a model with the coefficient of all predictors as zero (null model) and another model with all possible predictors (full model). This shows that the selected model is better at wilting estimation even when all the predictors are present in the model. The C-statistic was used to calculate model predictability based on the area under the receiver operating characteristics (ROC) curve (Huang and Ling, 2005). Option PREDPROBS computed wilting scores from the trained models using maximum likelihood estimation. The wilting scores with first highest probability were retained as the model-derived wilting scores. If the model-derived classification matched the visually rated scores, it was assumed to be a correct classification. This classification was used to create a classification accuracy matrix based on the following equation:


(A) $$A\,c\,c\,u\,r\,a\,c\,y=\frac{N\,o\,o\,f\,s\,a\,m\,p\,l\,e\,s\,c\,l\,a\,s\,s\,i\,f\,i\,e\,d\,c\,o\,r\,r\,e\,c\,t\,l\,y}{t\,o\,t\,a\,l\,s\,a\,m\,p\,l\,e\,s\,i\,n\,t\,h\,e\,s\,e\,t}\times 100$$

#### Second Probability and Nearest Score Classification for Ordinal Wilting Scores

For each visually rated wilting score, the corresponding model-derived wilting scores were estimated: one with the highest and another with second highest probability. The visually rated scores were then matched with both of the model-estimated wilting scores. If either of the two scores matched with the visually rated scores, it was assumed to be a correct classification. This classification was called the second probability method and was used to create a classification accuracy matrix using Eq. (A). Another method used to improve classification accuracy was the nearest score method. Along with matching the visually rated score with the corresponding model-derived score, two of the nearest values (the preceding and succeeding values) of the visually rated score were also matched. If any of the values (the actual value or the succeeding and preceding values) matched with the model-derived score, it was assumed to be a correct classification. This classification was further used to create a classification accuracy matrix using Eq. (A).

#### Cross-Validation

Leave-one-out cross-validation (CV) and k-fold CV were performed using Proc SURVEYSELECT. For the leave-one-out CV, each model was trained using N-1 data points (N being the number of images, 167 proximal and 84 aerial images) and the model was validated on the left-out data point. The process was repeated N times by iterating it in the loop function of SAS macros using each of the data points (the one data point that was randomly left out in each iteration) for validation every time. The C-statistic was performed to determine the area under the curve (AUC) value of each trained model. For k-fold CV, N data points were divided randomly into 10 sets (10-fold cross-validation). Each of the 10 sets consisted of 10% data points for validation and the remaining 90% for training the model. There was no overlapping among the validation sets. The C-statistic was performed on each of the 10 sets by iterating the models in the loop function of the SAS macros to determine the AUC value for each model (Stone, 1974; Hosmer et al., 2013).

#### Model Validation

The ordinal and logistic models derived in 2018 were validated using 2019 data. The RGB color indices derived from 2019 aerial and proximal images were replaced correspondingly in the trained models of 2018. Accuracy matrices were created for all models using the wilting values estimated using aerial indices of 2019.

## Results

### Wilting, Soil Moisture, and Pod Yield

**FIGURE 4:**
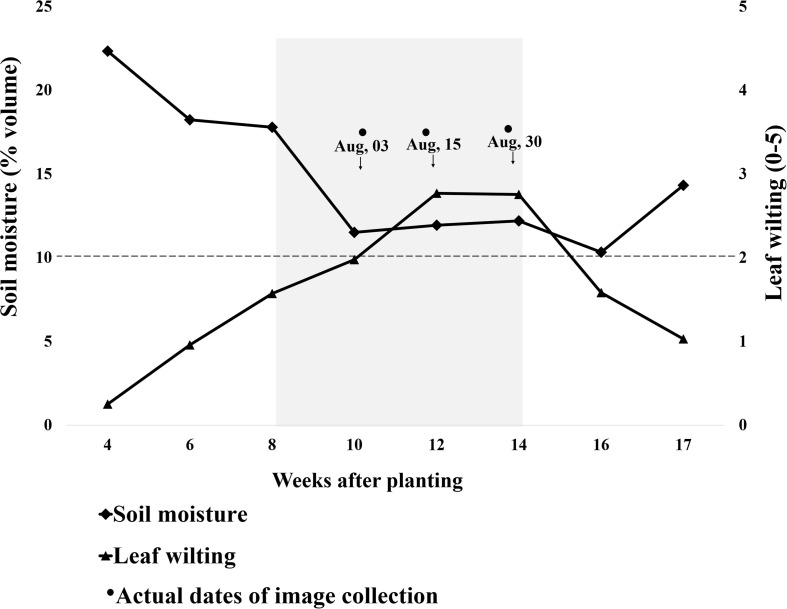
Progression of leaf wilting and soil volumetric water content (average of 0–30 cm depth) throughout the growing season. The *x*-axis represents weeks after planting (WAP); planting was done on May 17, 2018. The *y*-axis on the left represents soil moisture measured using a Delta-T HH2 moisture meter, and the right side is leaf wilting scored by visual estimation. The moisture meter was placed in one check plot per replication having genotype “Wynne,” and the soil moisture values are an average of all three replications. The wilting values are an average of all genotypes over three replications. Low soil moisture stress was artificially induced from 8 to 14 WAP. The gray shaded area represents the plots being covered by rainout shelters. Wilting scores above 2 (2 inclusive) were used as threshold for binary wilting score (turgid/wilted).

**FIGURE 5:**
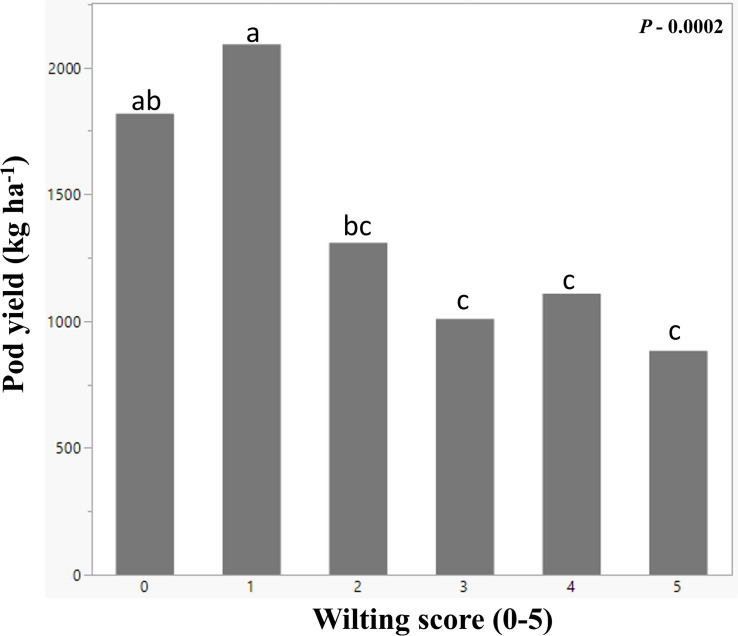
Mean peanut pod yield (*y-*axis) from peanut plots subjected to various degrees of leaf wilting (*x-*axis). The wilting scores were from 0 to 5, 0 being the healthy plant and 5 being the most wilted. The bars with same letters on the top are not significantly different using Fisher’s LSD at α = 0.05.

As soil moisture decreased, wilting increased (Figure 4). Before plots were covered, soil moisture slightly decreased and wilting increased in response to weekly rainfall variation, temperature increase, and plant growth and water use. Within 15 days from plot coverage, soil moisture at 30 cm depth dropped from 0.18 to 0.11 m^3^ m^–3^, continuing at this level until rainout shelters were removed and plots were uncovered. The average plot wilting score was raised from 1.6 to nearly 2 in the 2 weeks after plot coverage and to nearly 3 after two additional weeks, and it remained at that level until shelters were removed. Eight days after shelter removal, soil moisture slightly decreased from 0.12 to 0.10 m^3^ m^–3^ although the plants were less wilted, i.e., wilting score was 1.6 (Figure 4). On September 8 and 9, all plots received 3.2 mm of rainfall. From this point on, wilting was lessened and soil moisture increased so that, on September 18, at 17 WAP, average score was 1 and soil moisture was 0.14 m^3^ m^–3^ (Figure 4). No more data were taken as the plots were harvested on September 20. To evaluate the effect of soil moisture-induced wilting on peanut yield, plots within similar wilting scores were grouped and pod yield was averaged by wilting score (Figure 5). Wilting significantly (*p* = 0.0002) reduced pod yield from an average of 2000 kg ha^–1^ for plants scoring 0 and 1 to 880 kg ha^–1^ when they were severely wilted (score = 5) (Figure 5). Yield decreased by 33% when wilting was scored 2 as compared with 1 or 2 and by 49% when wilted plots from 3 to 5 were averaged. Wilting score 2 corresponded with 0.10 m^3^ m^–3^ volumetric soil water.

### RGB Color Indices and Relationships With Leaf Wilting

The wilting score corresponding to the proximal images collected on August 3 and 15 ranged from 0 to 3, whereas the scores corresponding to aerial images taken on August 30 ranged from 0 to 5. Overall, wilting was significantly correlated with all proximally and aerially derived RGB color indices (Table 3), but the greatest association (*p* < 0.0001) was for hue angle (*r* = −0.67, −0.66), a^∗^ (*r* = 0.70, 0.77), u^∗^ (*r* = 0.70, 0.74), GA (*r* = −0.61, −0.96), GGA (*r* = −0.68, −0.64), and CSI (*r* = 0.65, 0.63) (Table 3).

**TABLE 3:** Pearson correlation coefficients (*r*-value) of the RGB color indices measured proximally and aerially with visually rated leaf wilting for peanut genotypes.

RGB color indices	Proximal	Aerial
Intensity	0.27*	0.69
Hue	−0.67	−0.66
Saturation	−0.48	−0.76
Lightness	0.27*	0.48
a*	0.70	0.77
b*	−0.55	−0.65
u*	0.70	0.74
v*	−0.57	−0.53
Green area	−0.61	−0.69
Greener area	−0.68	−0.64
Crop senescence index	0.65	0.63

*Values not significant at *P* < 0.0001.

### Ordinal Logistic Models to Estimate Wilting (Ordinal 0–5 Rating)

The color space indices, selected using stepwise selection, were used as predictors in each model to generate probabilities for individual wilting scores. The wilting score with the highest probability and the maximum likelihood was retained as the image-derived score. The probabilities of wilting scores (0–5) were denoted as P_0_, P_1_, P_2_, P_3_, P_4_, and P_5_, respectively, where P_0_+P_1_+P_2_+P_3_ = 1 for model 1, and P_0_+P_1_+P_2_+P_3_+P_4_+P_5_ = 1 for model 2. P_4_ and P_5_ in model 1 (proximal data) were not generated because wilting scores 4 and 5 were absent on August 3 and 15 when proximal data were taken.

Probabilities and their formulas for each model are presented below.

Model 1 for proximal RGB images:


$$P_{0}=\frac{e^{\left(\varepsilon_{a}-11.75\right)}}{1+e^{\left(\varepsilon_{a}-11.75\right)}}$$

$$P_{1}=\frac{e^{\left(\varepsilon_{a}-7.19\right)}}{1+e^{\left(\varepsilon_{a}-7.19\right)}}-P_{0}$$

$$P_{2}=\frac{e^{\left(\varepsilon_{a}-4.28\right)}}{1+e^{\left(\varepsilon_{a}-4.28\right)}}-P_{0}-P_{1}$$

$$P_{3}=1-P_{0}-P_{1}-P_{2}$$

Model 2 for aerial RGB images:


$$P_{0}=\frac{e^{\left(\varepsilon_{b}+25.93\right)}}{1+e^{\left(\varepsilon_{b}+25.93\right)}}$$

$$P_{1}=\frac{e^{\left(\varepsilon_{b}+27.54\right)}}{1+e^{\left(\varepsilon_{b}+27.54\right)}}-P_{0}$$

$$P_{2}=\frac{e^{\left(\varepsilon_{b}+29.61\right)}}{1+e^{\left(\varepsilon_{b}+29.61\right)}}-P_{0}-P_{1}$$

$$P_{3}=\frac{e^{\left(\varepsilon_{b}+31.04\right)}}{1+e^{\left(\varepsilon_{b}+31.04\right)}}-P_{0}-P_{1}-P_{2}$$

$$P_{4}=\frac{e^{\left(\varepsilon_{b}+33.07\right)}}{1+e^{\left(\varepsilon_{b}+33.07\right)}}-P_{0}-P_{1}-P_{2}-P_{3}$$

$$P_{5}=1-P_{0}-P_{1}-P_{2}-P_{3}-P_{4},$$

where *e* = 2.718 is the Euler’s number,


$$\varepsilon_{a}=1.70\,u^{*}-1.77\times a^{*}-0.15\times C\,S\,I,a\,n\,d$$

$$\varepsilon_{b}=78.45\times i\,n\,t\,e\,n\,s\,i\,t\,y+6.96\times s\,a\,t\,u\,r\,a\,t\,i\,o\,n-1.08\times l\,i\,g\,h\,t\,n\,e\,s\,s$$

$$-2.62\times a^{*}+1.44\times u^{*}-45.09\times G\,G\,A-0.34\times C\,S\,I$$

### Binary Logistic Model to Estimate Wilting (Turgid vs. Wilted Rating)

Stepwise selected predictors with the first highest probability and maximum likelihood were used in models 3 and 4 to generate probabilities for binary wilting rating, i.e., P_*w*_ being the probability for a wilted plant and Pt the probability for a turgid plant where P_*w*_ + P_*t*_ = 1.

Model 3 for proximal RGB images:


$$P_{t}=\frac{e^{\left(\varepsilon_{c}-0.43\right)}}{1+e^{\left(\varepsilon_{c}-0.43\right)}}$$

$$P_{w}=1-P_{t}$$

Model 4 for aerial RGB images:


$$P_{t}=\frac{e^{\left(\varepsilon_{d}-31.74\right)}}{1+e^{\left(\varepsilon_{d}-31.74\right)}}$$

$$P_{w}=1-P_{t},$$

where *e* = 2.718 is the Euler’s number,


$$\varepsilon_{c}=11.70\times s\,a\,t\,u\,r\,a\,t\,i\,o\,n-0.07\times C\,S\,I,a\,n\,d$$

$$\varepsilon_{d}=-0.21\times l\,i\,g\,h\,t\,n\,e\,s\,s-0.65\times a^{{}^{*}}-37.90\times G\,G\,A-0.28\times C\,S\,I$$

Model 1 (proximal) with a^∗^, u^∗^, and CSI as selected predictors had AIC and SC values of 286.6 and 305.3 as compared with 376.4 and 385.7 for the null model (model with coefficient of predictors as 0), and 291.1 and 344.8 for the full model (model with all available predictors) (Table 4). Similarly, model 2 (aerial) with intensity, saturation, lightness, a^∗^, u^∗^, GGA, and CSI as predictors had AIC and SC values of 201.03 and 234.28, respectively, as compared with 344.2 and 358.1 for the null model, and 208.9 and 253.2 for the full model (Table 5). Based on the c-statistic, the area under the ROC curve (model predictability) was 0.83 for model 1 and 0.92 for model 2.

**TABLE 4:** Akaike information criterion (AIC) and Schwarz criterion (SC) values of the selected, full model (model with all available predictors), and null model (model with coefficient of predictors as 0) trained by ordinal and binary logistic regressions using color and vegetation indices from proximal and aerial RGB images.

		Ordinal logistic		Binary logistic	
			
Model		Proximal	Aerial	Proximal	Aerial
Selected	AIC	286.6	201.0	175.7	110.5
	SC	305.3	234.3	185.0	126.1
Full	AIC	291.1	208.9	184.3	104.4
	SC	344.8	253.2	221.7	141.9
Null	AIC	376.4	344.2	232.2	209.9
	SC	385.7	358.1	235.3	213.0

*A low value of AIC and SC signifies good statistical fit of the model.*

**TABLE 5:** Wilting accuracy matrix with the number of manually taken wilting scores (2018) on visual scale at the left and outside the table and the count of image-derived wilting scores in the table.

		Image-derived wilting score (0–5 scale) Proximal images					
	
Visual wilting score	Number of manually taken wilting scores	0	1	2	3	4	5
**0**	4	0	4	0	0	•	•
**1**	72	0	52	20	0	•	•
**2**	65	0	20	41	4	•	•
**3**	26	0	0	20	6	•	•
**4**	0	•	•	•	•	•	•
**5**	0	•	•	•	•	•	•
Total	**167**						
Accuracy	59%	0	72%	63%	23%	•	•
Accuracy (second probability method)	91%						
Accuracy (nearest score method)	99%						

		**Aerial images**					
	
**Visual wilting score**	**Number of manually taken wilting scores**	0	1	2	3	4	**5**
**0**	87	85	0	2	0	0	0
**1**	13	0	3	8	1	1	0
**2**	27	0	2	13	6	2	0
**3**	20	0	0	7	6	6	0
**4**	16	0	0	5	3	8	0
**5**	5	0	0	1	1	2	1
Total	**168**						
Accuracy	69%	98%	23%	48%	31%	50%	20%
Accuracy (second probability method)	81%						
Accuracy (nearest score method)	90%						

Wilting was on a scale of 0 to 5^†^. The percentage represents the fraction of wilting values that were estimated correctly using RGB color indices^‡^ derived from RGB images. Indices were used to estimate leaf wilting using ordinal logistic regression*. The proximal images were taken 11 and 13 weeks after planting (WAP), whereas the aerial images were taken 15 WAP.

*^†^A score of 0 represents potentially healthy plant with no wilting or leaf drooping symptoms; 1 represents some terminal and newer leaves fold up but overall the plant looks healthy; 2 represents an almost all leaves fold up and show signs of wilting, lower and older leaves start to fold; 3 represents wilting and drooping shows up on all leaves of the plant, low-moisture effect on older leaves is more prominent, bare ground starts to show up under the plant as leaves wilt; 4 represents all leaves are wilted and some leaves start to change color due to chlorophyll degradation, bare ground is prominently visible, some leaves have dried and crisped up; 5 represents all leaves have severely wilted and color of all leaves is light green to yellow, bare ground is fully visible, more than 50% of leaves are crisp and dry, the plant is almost physiologically dead.*

^‡^RGB color indices – Intensity, Hue, Saturation, Lightness, a*, b*, u*, v*, green area (GA), greener area (GGA), crop senescence index (CSI).

*Allison, 2012.

**FIGURE 6:**
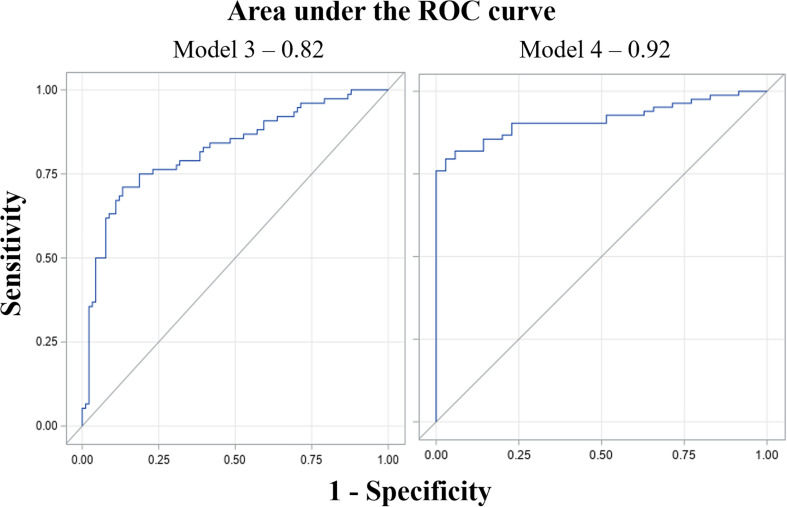
Receiver operating characteristic (ROC) curves of model 3 (proximal data) and model 4 (aerial data) for estimating irrigation regimes. The x-axis is false positive rate (1-specificity) and the y-axis is true positive rate (sensitivity). The area under the curve (AUC) value (0.82 and 0.92 for the models) represents predictability of the models. AUC value ranges from 0 to 1; 0 being 100% predictions of the model are wrong; whereas 1 means 100% of predictions made by the model are right (Huang and Ling, 2005).

Model 3 (proximal) with saturation and CSI as predictors had AIC and SC values of 175.7 and 185.0 as compared with 232.2 and 235.3 for the null model and 184.3 and 221.7 for the full model (Table 4). Model 4 (aerial) with lightness, a^∗^, GGA, and CSI as predictors had AIC and SC values of 110.5 and 126.1, respectively, as compared with 209.9 and 213.0 for the null model and 104.4 and 141.9 for the full model (Table 4). Based on the c-statistic, the area under the ROC curve was 0.82 for model 3 and 0.92 for model 4 (Figure 6).

### The Classification Accuracy

The classification accuracy of the model-derived wilting (for ordinal 0–5 score) was 69% for aerial and 61% for proximal data (Table 5). The accuracy increased to 81% for aerial and 91% for proximal when using the second highest probability and to 90% for aerial and 99% for proximal when using the nearest score method. The classification accuracy of the model-derived wilting (for binary turgid vs. wilted rating) was 88% for aerial and 77% for proximal (Table 6). Correlating the ordinal and binary wilting scores taken manually with derived ones, aerially derived wilting had *r*-values of 0.85 for ordinal and 0.71 for binary, whereas proximally derived wilting had *r*-values of 0.60 for ordinal and 0.54 for binary (data not shown).

**TABLE 6:** Wilting accuracy matrix with the number of manual wilting scores (2018) on visual scale at the left and outside the table and the count of image-derived wilting scores in the table.

	Estimated turgid vs. wilted plants					
	
	Proximal images			Aerial images		
Plant water status	No of plots within each water status	**Turgid**	**Wilted**	No of plots within each water status	**Turgid**	**Wilted**
**Turgid**	76	55	21	116	106	10
**Wilted**	91	17	74	52	11	41
Total	**167**			**168**		
Accuracy	77%	72%	81%	88%	91%	79%

Wilting was on a binary scale of turgid/wilted^†^. The percentage represents the fraction of wilting scores that were estimated correctly using RGB color indices^‡^ derived from RGB images. Indices were used to estimate leaf wilting using ordinal logistic regression*. The proximal images were taken 11 and 13 weeks after planning (WAP) whereas the aerial images were taken 15 WAP.

*^†^Wilting score 0 and 1 were rated as turgid and scores above 2 (2 inclusive) were rated as wilted.*

^‡^RGB color indices – Intensity, Hue, Saturation, Lightness, a*, b*, u*, v*, green area (GA), greener area (GGA), crop senescence index (CSI).

*Allison, 2012.

### Cross-Validation

The mean AUC of the ROC curve derived from the 10-fold cross-validation was 0.83 for model 1, 0.93 for model 2, 0.82 for model 3, and 0.93 for model 4 (Table 7). The mean AUC values using leave-one-out cross-validation was 0.83 for model 1, 0.92 for model 2, 0.82 for model 3, and 0.93 for model 4 (Table 7).

**TABLE 7:** Area under the receiver operating characteristic (ROC) curve derived using c-statistic for 10-fold cross-validation sets.

		Proximal			Aerial	
				
Folds	*N*	Ordinal	Binary	*N*	Ordinal	Binary
1	16	0.83	0.82	16	0.92	0.94
2	16	0.83	0.82	16	0.93	0.93
3	16	0.83	0.81	17	0.92	0.93
4	17	0.84	0.83	17	0.93	0.94
5	17	0.82	0.82	17	0.92	0.93
6	17	0.83	0.81	17	0.93	0.93
7	17	0.83	0.82	17	0.92	0.94
8	17	0.84	0.83	17	0.94	0.93
9	17	0.83	0.85	17	0.92	0.92
10	17	0.82	0.82	17	0.92	0.93
Total	167			168		
Mean		0.83	0.82		0.93	0.93

*N is the number of data points assigned to the validation set for that fold. The model used for the c-statistic on each validation set was trained using the remaining (167 – N for proximal, and 168 – N for aerial) data points. All 10-fold sets were randomly generated.*

### Model Validation

Models validated using 2019 RGB color indices and wilting data showed classification accuracy of 71% using model 1 and 75% using model 2 (Table 8). The classification accuracy increased to 91% and 94% using the second probability method and 96% and 98% using the nearest score method. Classification accuracy for binary logistic models were 93% for model 3 and 95% for model 4.

**TABLE 8:** Wilting accuracy matrix with the number of manually taken wilting scores (2019) on visual scale at the left and outside the table and the count of model estimated wilting scores in the table.

	**Image-derived wilting score (0–5 scale) Proximal images**						
	
**Visual wilting score**	**Number of manually taken wilting scores**	**0**	**1**	**2**	**3**	**4**	**5**
**0**	71	69	0	0	2	0	0
**1**	12	10	0	0	2	0	0
**2**	14	1	0	0	12	1	0
**3**	49	1	0	0	42	6	0
**4**	21	0	0	0	14	7	0
**5**	0	•	•	•	•	•	•
Total	**167**						

Accuracy	71%	97%	0	0	86%	33%	
Accuracy (second probability method)	91%						
Accuracy (nearest score method)	96%						

	Aerial images						
	
Visual wilting score	Number of manually taken wilting scores	0	1	2	3	4	5
**0**	72	71	0	1	0	0	0
**1**	12	11	0	1	0	0	0
**2**	14	0	0	3	11	0	0
**3**	49	2	0	1	43	3	0
**4**	21	0	0	0	12	9	0
**5**	0	•	•	•	•	•	•
Total	**168**						
Accuracy	75%	99%	0%	21%	88%	43%	•
Accuracy (second probability method)	94%						
Accuracy (nearest score method)	98%						

*Wilting was on a scale of 0 to 5^†^. The percentage represents the fraction of wilting values from 2019 that were estimated correctly using the logistic model derived in 2018. The 2018 logistic models were validated by substituting the RGB color indices^‡^ values derived in 2019. The proximal and aerial images were taken at 15 weeks after planting.*

*^†^A score of 0 represents a potentially healthy plant with no wilting or leaf drooping symptoms; 1 represents some terminal and newer leaf fold-up, but overall the plant looks healthy; 2 represents almost all leaves folded up and show signs of wilting, lower and older leaves start to fold; 3 represents wilting and drooping shows up on all leaves of the plant, low-moisture effect on older leaves is more prominent, bare ground starts to show up under the plant as leaves wilt; 4 represents all leaves are wilted and some leaves start to change color due to chlorophyll degradation, bare ground is prominently visible, some leaves have dried and crisped up; 5 represents all leaves have severely wilted, and color of all leaves is light green to yellow, bare ground is fully visible, more than 50% of leaves are crisp and dry, the plant is almost physiologically dead.*

^‡^RGB indices – Intensity, Hue, Saturation, Lightness, a*, b*, u*, v*, green area (GA), greener area (GGA), crop senescence index (CSI).

## Discussion

The 28 genotypes used in this study represent a subset of the United States mini-core peanut germplasm collection with contrasting morphological and physiological traits and with different responses to water deficit stress (Holbrook et al., 1993; Holbrook and Dong, 2005). In response to induced low soil moisture stress by covering the plots with rainout shelters from 8 to 14 WAP, the genotypes were visibly wilted with wilting scores ranging from 0 to 3 after four and from 0 to 5 after 6 weeks of stress (Figure 4). The beginning of induced moisture stress treatment coincided with beginning pegging and pod growth stages, which are growth stages sensitive to moisture stress with significant effects on yield (Rowland et al., 2007). Indeed, low soil moisture decreased peanut yield from an expected 4500 kg ha^–1^ to only 2000 kg ha^–1^ or less, which corresponds with peanut production in Virginia (Balota et al., 2020). For example, year 2014 was a good peanut year with a state average of 5040 kg ha^–1^, but year 2010 was dry, and average yield was only 2016 kg ha^–1^ (US Department of Agriculture-National Agricultural Statistics Service, 2019). This is because, under drought, chlorophyll content, carbon assimilation and photosynthetic efficiency decrease, and ultimately, biomass and yield accumulation are less (Reddy et al., 2003; Pilon et al., 2018). In this study, plots that maintained turgid vines, i.e., genotypes scored 0 and 1, had the highest yield under drought. The plots for which wilting was scored 2–5 produced only around 1000 kg ha^–1^ with a 30% initial yield decrease when wilting was scored 2 followed by 50% yield reduction for plots with greater wilting (Figure 5). If the relationship between plant wilting and yield is proven on larger-scale production, a wilting score of 2 could be used to trigger irrigation of peanut, and aerial estimations could become important for efficient irrigation scheduling as suggested by Rowland et al. (2012).

The RGB-derived color indices from proximal and aerial images were associated with leaf wilting (Table 3). For example, due to water deficit, canopy color shifted from green (120°) to yellow (60°), i.e., chlorophyll content decreased, resulting in lower hue angles in wilted plants than turgid plants. Similarly, severely wilted plants had a smaller fraction of green pixels captured with canopy images and, therefore, less GA and GGA. The indices a^∗^ and u^∗^ increased with wilting increase; they became less negative, which indicates that canopy color changed from green to red in stressed and wilted plants. Similar results were found in soybean, wheat, corn, and peanuts confirming our hypothesis that RGB images can discriminate turgid vs. wilted peanut canopies, i.e., tissue turgor pressure falls close to zero under low soil moisture stress (Vergara-Díaz et al., 2015, 2016; Balota and Oakes, 2017; Zhou et al., 2020).

Wilting models 1 and 2 had classification accuracies of 61% for proximal and 69% for aerial images (Table 5). The accuracy improved when using the first and second highest probability for classification to 91% for proximal images and 81% for aerial images. When using the nearest score classification, wilting estimation accuracy raised to 99% for proximal images and 90% for aerial images (Table 5). Lower accuracy from aerial images could be attributed to reduced resolution in comparison with proximal images. Another reason could be the number of wilting levels, which were fewer for proximal imagery (wilting level 4 and 5 were absent) as compared with aerial images. Nonetheless, when using the nearest score classification, the accuracy of wilting estimation from aerial images increased substantially. This method is also the closest to the traditional method of visual rating. For example, when visually rating, the operator may find it difficult to assign a plot clearly to a score of 1 or 2 or rather in between 1 and 2. If rating is being performed by different operators, i.e., different visual perceptions, or at different hours of the day, i.e., different sun angles can influence visual perception, then a plot that scores 2 may be assigned 1 or 3. The wilting matrix confirmed that the majority of misclassifications were neighbors, e.g., either 2 or 4 for a score of 3. In addition, the statistical probabilities showed that the wilting scores assigned on the basis of the first highest probability were marginally ahead of the second highest probability. For example, for plot 121, the score 2 had first highest probability of 0.37, whereas the second highest probability was 0.35 for score 1 (data not shown). Therefore, using either the first or second highest probability as a true classification or nearest score classification to calibrate the process and estimate wilting scores is closer to the visual rating and had improved accuracy. The AUC values for model 1 (proximal) (0.83) and model 2 (aerial) (0.92) also support the conclusion that these models are highly predictive of peanut plant wilting. Though aerially derived wilting scores had lower classification accuracy, they correlated better (*r* = 0.85) and had higher predictability (AUC = 0.92) than proximally derived wilting scores (*r* = 0.60; AUC = 0.83) (correlation data not shown). Aerial estimation was preferred to proximal for faster data collection and analysis. It was found in this study that aerial imagery can be accomplished in a shorter period of time than proximal imaging (a 12-min flight for aerial imagery compared to 90 min for proximal imagery for 168 peanut plots), and rating using aerial sensing offers greater temporal repeatability. Although aerial imagery involves processing time (about 5 h) to orthomosaic the image, the process is fully automated at night and does not require personnel time. Moreover, manually taken proximal images were prone to errors, such as differences in camera height, sun angle, and cloud cover during the time of collection. These drawbacks were absent in aerial imagery.

Wilting models 3 (binary proximal) and 4 (binary aerial) had classification accuracies of 77% for proximal images and 88% for aerial imagery (Table 6). The AUC for model 3 was 0.82 and for model 2 was 0.87 (Figure 6). Although classification accuracy and AUC were both used for learning algorithms in machine learning, AUC is considered better and statistically more consistent than classification accuracy (Ling et al., 2003; Huang and Ling, 2005). Thus, based on AUC values, the probability of these models to estimate wilting rating was 82% when using proximal imagery and 92% when using aerial imagery. Moreover, aerially derived wilting scores correlated better with manually taken wilting scores (*r* = 0.71) than proximally derived wilting scores (*r* = 0.54) (data not shown). Aerial sensing to estimate soil moisture and trigger irrigation of crops is currently used (Ahmad et al., 2009; Ge et al., 2019). For peanut, however, with dense biomass growth and complete ground coverage early in the growing season, similar approaches may not work. Alternatively, using the binary wilting rating (turgid vs. wilted) estimated by models 3 and 4 can be used to represent plant water status. This has the potential for development of smartphone applications that can be used to decide if a peanut field requires irrigation or not, i.e., plants having turgid rating would not require irrigation, but wilted plants would require irrigation. However, this needs further investigation and feasibility analysis for implementation in peanut production.

All logistic regression models used here have high values of the mean AUC associated with cross-validation, i.e., 0.83 for model 1, 0.93 for model 2, 0.82 for model 3, and 0.93 for model 4 (Table 7). This proves the robustness of the models when used on independent data sets. Using 2019 data for validation of all models resulted in similar wilting classification accuracies as in 2018 (Tables 8, 9). This shows that the wilting estimation models developed in this study can be successfully used with other data sets.

**TABLE 9:** Wilting accuracy matrix with the number of manual wilting scores (2019) on the visual scale at the left and outside the table and the count of image-derived wilting scores in the table.

	Estimated turgid vs. wilted plants					
	
	Proximal images			Aerial images		
		
Plant water status	No of plots within each water status	Turgid	Wilted	No of plots within each water status	Turgid	Wilted
**Turgid**	89	82	7	90	86	4
**Wilted**	78	5	73	78	5	73
Total	**167**			**168**		
Accuracy	93%	92%	94%	95%	96%	94%

*Wilting was on a binary scale of turgid/wilted^†^. The percentage represents the fraction of wilting values that were estimated correctly using logistic model derived in 2018. The 2018 binary models were validated by substituting the RGB color indices^‡^ values derived in 2019. The proximal and aerial images were taken at 15 weeks after planting.*

*^†^Wilting score 0 and 1 were rated as turgid and scores above 2 (2 inclusive) were rated as wilted.*

^‡^Color space indices – Intensity, Hue, Saturation, Lightness, a*, b*, u*, v*, green area (GA), greener area (GGA), crop senescence index (CSI).

In this article, we report new models to predict wilting in peanut and a step forward toward future automation and real-time selection for reduced wilting and improved yield and drought tolerance in the peanut breeding programs in the United States and throughout the word. We also report, for the first time, prediction models for peanut plant water status that can be useful for irrigation scheduling in the future. For example, currently, only 5% of the remote sensing models proposed to schedule irrigation are implemented at the farm level in the United States (Steve Thomason, personal communication). Our wilting approach from RGB images could be more appealing for growers to use than multispectral sensors as in other proposals, but this needs further investigation at the farm level. In a previous work, we document substantial time savings when using high-throughput techniques in breeding (Sarkar et al., 2020). At the same time, high-throughput techniques, such as reported in this article, can increase the frequency of data collection and possibly the accuracy by removing the bias of visual human perception (Milberg et al., 2008; Borra-Serrano et al., 2018; Sarkar and Jha, 2020). Overall, the logistic models developed in this study were successful in estimating peanut wilting and plant water status from RGB images, which suggests potential for aerial imagery and machine learning applications in breeding for improved drought tolerance of peanut.

## Conclusion

This article reports, for the first time, on a simple yet key aspect of peanut screening for tolerance to low soil moisture stress and uses novel, fast, cost-effective, and accurate RGB-derived models to estimate leaf wilting. Leaf wilting caused by low soil moisture stress can be estimated using remote sensing. It can also be used to predict plant water status associated with maximum yield. An important aspect of this study is the combined use of remote sensing and machine learning tools to build models for high-throughput phenotyping. These methods are helpful in quick and accurate data collection in research and breeding programs and suggest good scope for further investigation of automated and efficient irrigation in peanut production.

## Data Availability Statement

The software presented in this article are not readily available because of proprietary access. Requests to access the software should be directed to the corresponding author. The raw data supporting the conclusions of this article will be made available by the authors, without undue reservation.

## Author Contributions

MB wrote the grant proposal, selected the peanut genotypes to be used, and had substantial contribution at the reviewing and editing of the manuscript. A-BC helped developing protocols and routines for image collection, processing, and analysis. AR helped with the regression analysis and developing the logistic models for this study. SS did the image and data collection, their analysis, and wrote the manuscript. All authors contributed to the article and approved the submitted version.

## Conflict of Interest

The authors declare that the research was conducted in the absence of any commercial or financial relationships that could be construed as a potential conflict of interest.

## Footnotes

1
http://github.com/george-haddad/CIMMYTopensoftware

2
http://fiji.sc/Fiji

## Abbreviations

AIC: Akaike Information Criterion
AUC: area under the curve
CSI: crop senescence index
CV: cross validation
GA: green area
GGA: greener area
RGB: red-green-blue
ROC: receiver operating characteristic
SC: Schwarz Criterion.
