# Corrigendum: Peanut Leaf Wilting Estimation From RGB Color Indices and Logistic Models

**DOI:** 10.3389/fpls.2021.821325

**Published:** 2022-01-05

**Authors:** Sayantan Sarkar, A. Ford Ramsey, Alexandre-Brice Cazenave, Maria Balota

**Affiliations:** ^1^School of Plant and Environmental Sciences, Virginia Tech, Tidewater AREC, Suffolk, VA, United States; ^2^Department of Agricultural and Applied Economics, Virginia Tech, Blacksburg, VA, United States

**Keywords:** peanut leaf wilting, RGB color space indices, logistic regression, machine learning, high-throughput phenotyping

In the original article, there was a mistake in Table 5 as published. There were typos in the text and numbers of the table. The corrected Table 5 appears below:

**Table 5:** Wilting accuracy matrix with the number of manually taken wilting scores (2018) on a visual scale at the left and outside the table and the count of image-derived wilting scores in the table.

		**Image-derived wilting score (0–5 scale)**					
		**Proximal images**					
**Visual wilting score**	**Number of manually taken wilting scores**	**0**	**1**	**2**	**3**	**4**	**5**
**0**	4	0	4	0	0	∙	∙
**1**	72	0	52	20	0	∙	∙
**2**	65	0	20	41	4	∙	∙
**3**	26	0	0	20	6	∙	∙
**4**	0	∙	∙	∙	∙	∙	∙
**5**	0	∙	∙	∙	∙	∙	∙
Total	**167**						
Accuracy	59%	0	72%	63%	23%	∙	∙
Accuracy (second probability method)	91%						
Accuracy (nearest score method)	99%						
		**Aerial images**					
**Visual wilting score**	**Number of manually taken wilting scores**	**0**	**1**	**2**	**3**	**4**	**5**
**0**	87	85	0	2	0	0	0
**1**	13	0	3	8	1	1	0
**2**	27	0	2	13	6	2	0
**3**	20	0	0	7	6	6	0
**4**	16	0	0	5	3	8	0
**5**	5	0	0	1	1	2	1
Total	**168**						
Accuracy	69%	98%	23%	48%	31%	50%	20%
Accuracy (second probability method)	81%						
Accuracy (nearest score method)	90%						

Wilting was on a scale of 0 to 5. The percentage represents the fraction of wilting values that were estimated correctly using RGB color indices derived from RGB images. Indices were used to estimate leaf wilting using ordinal logistic regression*. The proximal images were taken 11 and 13 weeks after planting (WAP) whereas the aerial images were taken 15 WAP.

*^†^A score of 0 represents potentially healthy plant with no wilting or leaf drooping symptoms; 1 represents some terminal and newer leaves fold up but overall, the plant looks healthy; 2 represents almost all leaves fold up and show signs of wilting, lower and older leaves start to fold; 3 represents wilting and drooping shows up on all leaves of the plant, low-moisture effect*.

In the original article, there was an error. There were typos in the equations of Model 1.

A correction has been made to **Results**, **Ordinal Logistic Models to Estimate Wilting (Ordinal 0-5 Rating)**, paragraph 3, Model 1 equations:

Model 1 for proximal RGB images:


$$\begin{array}{l} P_{0}=\;\frac{e^{\left(\varepsilon_{a\;}-\;11.75\right)\;}}{1+\;e^{\left(\varepsilon_{a\;}\;-\;11.75\right)}} \end{array}$$

$$\begin{array}{l} P_{1}=\;\frac{e^{\left(\varepsilon_{a\;}\;-\;7.19\right)\;}}{1+\;e^{\left(\varepsilon_{a}-\;7.19\right)}}-P_{0} \end{array}$$

$$\begin{array}{l} P_{2}=\;\frac{e^{\left(\varepsilon_{a\;}-\;4.28\right)\;}}{1+\;e^{\left(\varepsilon_{a}-\;4.28\right)}}-P_{0}-P_{1} \end{array}$$

$$\begin{array}{l} P_{3}=\;1-P_{0}-P_{1}-P_{2} \end{array}$$

In the original article, there was a mistake in Table 9 as published. There were typos in the numbers of the table. The corrected Table 9 appears below.

**Table 9:** Wilting accuracy matrix with the number of manual wilting scores (2019) on a visual scale at the left and outside the table and the count of image-derived wilting scores in the table.

	**Estimated turgid vs. wilted plants**					
	**Proximal images**			**Aerial images**		
**Plant water status**	**No of plots within each water status**	**Turgid**	**Wilted**	**No of plots within each water status**	**Turgid**	**Wilted**
**Turgid**	89	82	7	90	86	4
**Wilted**	78	5	73	78	5	73
Total	**167**			**168**		
Accuracy	93%	92%	94%	95%	96%	94%

*Wilting was on a binary scale of Turgid/Wilted. The percentage represents the fraction of wilting values that were estimated correctly using the logistic model derived in 2018. The 2018 binary models were validated by substituting the RGB color indices^‡^ values derived in 2019. The proximal and aerial images were taken 15 weeks after planting*.

*^†^Wilting scores 0 and 1 were rated as turgid and scores above 2 (2 inclusive) were rated as wilted*.

^‡^Color space indices – Intensity, Hue, Saturation, Lightness, a*, b*, u*, v*, green area (GA), greener area (GGA), crop senescence index (CSI).

The authors apologize for these errors and state that this does not change the scientific conclusions of the article in any way. The original article has been updated.

## Publisher's Note

All claims expressed in this article are solely those of the authors and do not necessarily represent those of their affiliated organizations, or those of the publisher, the editors and the reviewers. Any product that may be evaluated in this article, or claim that may be made by its manufacturer, is not guaranteed or endorsed by the publisher.

